# Tryptophan attenuates acute hypoxic stress-induced intestinal injury through the modulation of intestinal barrier integrity and gut microbiota homeostasis

**DOI:** 10.1016/j.gendis.2025.101627

**Published:** 2025-04-04

**Authors:** Jianhua Zheng, Jingqing Chen, Wensheng Zhang, Yunpeng Wu, Tongtong Qin, Yunzhi Fa, Qianyan Dong, Rui Zhang, Yefeng Qiu

**Affiliations:** a Academy of Military Medical Sciences, Beijing 100071, China; b Laboratory of Advanced Biotechnology, Beijing 100071, China

There is growing evidence that acute hypoxia can be hazardous to health by causing damage to a variety of human organs. Gastrointestinal dysfunction is a common symptom of acute mountain sickness, and the pathogenesis of acute hypoxic gastrointestinal injury is complex. Additionally, the incidence of gastrointestinal disorders in people travelling to highland areas increases with altitude.^1^ Studies have shown that tryptophan possesses stress-relieving effects, but the specific effects and mechanisms of tryptophan require further elucidation. In this study, we analyzed the potential mechanism of tryptophan in ameliorating hypoxic intestinal stress injury under the induction of acute hypoxic stress using scanning electron microscopy, 16S rDNA sequencing, and gas chromatography. Our results showed that tryptophan supplementation could promote the recovery of the intestinal barrier, reduce intestinal permeability, and increase the species diversity of gut microbiota. Furthermore, it promoted the growth of beneficial bacteria and the production of their functional metabolites, short-chain fatty acids, in hypoxic stress mice. This could effectively alleviate hypoxia-induced intestinal injury and improve intestinal health.

Erythrocytosis is one of the classical responses to high altitude, and thus we assessed the hematological parameters in mice subjected to 72 h of hypoxia using routine blood tests.^2^ Acute hypoxic stress for 72 h significantly decreased mouse body weight, erythrocyte counts, hemoglobin concentration, and erythrocyte specific volume (Fig. 1A, i–iv) were all significantly elevated (*p* < 0.05). However, this did not affect leukocyte and platelet counts (Fig. 1A–v, vi), which agreed with the previous study.^2^ This indicates the successful construction of the normobaric hypoxia model. Our previous study showed that 72 h of acute hypoxia can successfully establish a mouse model of hypoxic stress intestinal injury. Previous studies reported that 0.40% tryptophan was the most effective in alleviating intestinal injury.^3^ On this basis, the study of tryptophan alleviation of acute hypoxic intestinal stress injury was carried out by feeding high tryptophan content feed (0.40%).

**Figure 1:**
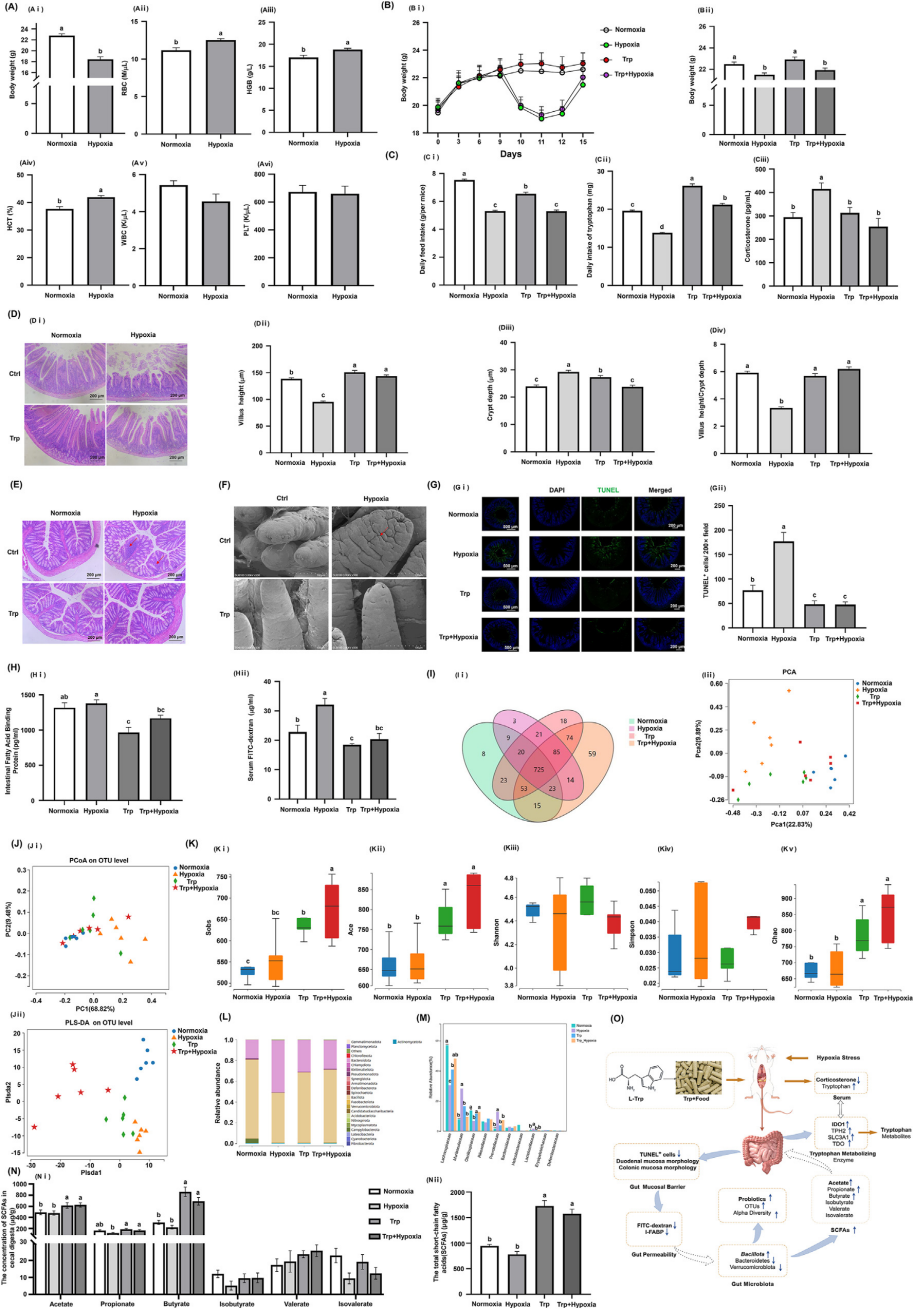
Tryptophan attenuates acute hypoxic stress-induced intestinal injury through the modulation of intestinal barrier integrity and gut microbiota homeostasis. **(A)** The changes in body weight and routine blood test indices including erythrocyte count, hemoglobin concentration, erythrocyte specific volume, leukocyte, and platelet counts of mice subjected to hypoxic stress for 72 h. **(B)** The effect of tryptophan on the body weight of mice subjected to hypoxic stress, the curve of body weight change during the experiment, and the difference in body weight at the end of the experiment. **(C)** The effect of tryptophan on average daily feed intake, daily tryptophan intake, and corticosterone hormone levels analyzed with ELISA, in hypoxic-stressed mice. **(D)** The effect of tryptophan on the morphology of the duodenal in hypoxia-stressed mice analyzed with hematoxylin-eosin staining. Image J software was used to measure and analyze the length of duodenal villi, the depth of crypts, and the ratio of villi length to crypt depth. **(E)** The effect of tryptophan on the morphology of the colonic in hypoxic-stressed mice analyzed with hematoxylin-eosin staining. **(F)** The effect of tryptophan on the morphology of duodenal villi in hypoxic-stressed mice analyzed with scanning electron microscopy. **(G)** The effect of tryptophan on apoptosis of intestinal epithelial cells in hypoxia-stressed mice, detection of apoptosis in mouse duodenal epithelial cells by terminal deoxynucleotidyl transferase dUTP nick-end labeling (TUNEL) staining, and counting of TUNEL-positive cells. **(H)** The effect of tryptophan on intestinal permeability in hypoxia-stressed mice, serum intestinal fatty acid binding protein and serum FITC-dextrose levels in that order. **(I)** The effect of tryptophan on principal component analysis (PCA) and Venn diagram distribution in hypoxic-stressed mice. **(J)** The effect of tryptophan on PCA of gut microbiota in hypoxia-stressed mice, principal coordinate analysis (PCoA), and partial least squares discriminant analysis (PLS-DA). **(K)** The effect of tryptophan on the Alpha diversity of gut microbiota induced by hypoxic stress in mice, including Sobs, Ace, Shannon, Simpson, and Chao1 indices. **(L)** The changes in mouse gut microbiota at the phylum level. **(M)** The effect of tryptophan on differences in relative abundance levels between gut microbiota at the family level. **(N)** The effect of tryptophan on the intestinal short-chain fatty acid content of acetate, propionate, butyrate, lsobutyrate, valerate, isovalerate, and the total short-chain fatty acid content in hypoxic-stressed mice. **(O)** Potential mechanisms for the protective effect of tryptophan on acute hypoxic stress-induced intestinal injury. “Normoxia” is the normoxic control group, “Hypoxia” is the hypoxic stress group, “Trp” is the tryptophan-supplemented group, and “Trp + Hypoxia” is the tryptophan-supplemented and hypoxic stress group.

Initially, this study analyzed the impact of tryptophan supplementation on body weight, food intake, stress hormone levels, and serum amino acid profiles in a murine model of hypoxia-induced stress. Tryptophan supplementation slowed down acute hypoxic stress-induced body weight loss, with body weight recovery being faster after the cessation of stress. However, the differences did not reach a significant level (Fig. 1B–i, ii). In addition, acute hypoxic stress induced a decrease in feed intake (*p* < 0.05), and the daily intake of tryptophan in tryptophan-supplemented mice was significantly higher than that in normoxic and hypoxic stress mice (*p* < 0.05) (Fig. 1C–i, ii). The results showed that tryptophan significantly reduced corticosterone hormone levels in mice with acute hypoxic stress (*p* < 0.05) (Fig. 1C–iii). Additionally, the serum tryptophan content of mice rose after tryptophan supplementation (Table S1), and the serum tryptophan content of hypoxic stress mice also rose.

The effects of tryptophan on the barrier function of intestinal in hypoxia-stressed mice were further investigated by hematoxylin-eosin staining, scanning electron microscopy, and terminal deoxynucleotidyl transferase dUTP nick-end labeling (TUNEL) staining. It was found that the duodenal villi of mice under acute hypoxic stress all showed shortening and shedding phenomena; the villus length was shortened, the crypt depth was increased, and the ratio of villus length to crypt depth was decreased (*p* < 0.05) (Fig. 1D–i–iv). The tryptophan significantly alleviated the duodenal injury induced by acute hypoxic stress. Moreover, the tryptophan alleviated the acute hypoxic stress-induced colonic inflammatory cell infiltration and crypt structure abnormalities (Fig. 1E). Scanning electron microscopy results showed that acute hypoxic stress resulted in damage to the duodenum, partial atrophy of the villi surface, and widening of the interstices. The tryptophan significantly alleviated this damage (Fig. 1F). TUNEL staining showed a significant reduction in apoptosis after tryptophan supplementation (*p* < 0.05) (Fig. 1G–i, ii).

To further evaluate the effect of tryptophan on intestinal injury and intestinal permeability in hypoxic mice, we measured serum intestinal fatty acid-binding protein (I-FABP) using ELISA and assessed intestinal permeability by orally administering the fluorescent marker FITC-dextran. Tryptophan significantly reduced serum I-FABP levels in mice subjected to acute hypoxic stress (*p* < 0.05) (Fig. 1H and i), indicating decreased intestinal injury. Tryptophan-supplemented mice showed a significant decrease in serum FITC-dextran levels (*p* < 0.05). This suggests that tryptophan supplementation significantly could attenuate the acute hypoxic stress-induced increase in intestinal permeability in mice (Fig. 1H and ii).

Tryptophan is a biosynthetic precursor of many metabolites and can enhance the barrier function of the intestinal through bioactive substances, which are produced by its own metabolism or microbial metabolism.^4^ We measured the expression levels of genes involved in tryptophan metabolizing enzymes in duodenal and colonic tissues. Tryptophan significantly increased the gene expression of indoleamine 2,3-dioxygenase 1 (*IDO1*), tryptophan hydroxylase 2 (*TPH2*), and solute carrier family 3 member 1 (*SLC3A1*) in duodenal tissues and that of *TPH2*, *TDO1*, and *SLC3A1* in colonic tissues of hypoxic-stressed mice (*p* < 0.05) (Figs. S1A–G). Hypoxia-induced stress may disrupt tryptophan metabolism, potentially leading to higher serum tryptophan levels in hypoxic mice compared with normoxic mice due to accumulation.

To gain insight into the effects of tryptophan on the gut microbiota of hypoxia-stressed mice, we analyzed the results using 16S rDNA sequencing. The tryptophan-supplemented group exhibited a greater number of operational taxonomic units compared with the hypoxic stress group (Fig. 1I–i). Principal component analysis revealed that the gut microbiota of mice in the hypoxic stress group and the tryptophan-supplemented hypoxic stress group were significantly differentiated (Fig. 1I–ii). Principal coordinate analysis based on the Bray–Curtis distance matrix demonstrated that the gut microbiota of mice in the hypoxic stress group was significantly different from the other three groups (Fig. 1J–i). Partial least squares discriminant analysis showed that the gut microbiota of mice from different groups could be distinguished, indicating compositional differences (Fig. 1J–ii). Tryptophan significantly increased the Sobs, Ace, and Chao1 indices of the fecal microbiota of mice (*p* < 0.05) (Fig. 1K–ii–v). At the phylum level, tryptophan primarily affected the relative abundance of *Bacillota*, *Bacteroidetes*, and *Campylobacterota* (Fig. 1L). Regarding the dominant species at the family level, tryptophan significantly increased the mean relative abundance of *Lachnospiraceae* and *Oscillospiraceae*, and significantly decreased that of *Muribaculaceae*, *Prevotellaceae*, and *Campylobacterota* in the intestinal tracts of hypoxia-stressed mice (*p* < 0.05) (Fig. 1M). Collectively, these results suggest that tryptophan supplementation could modify the gut microbiota structure in hypoxic-stressed mice, enhance species diversity, and foster the growth of beneficial bacteria, which may be a crucial factor in ameliorating the dysbiosis induced by hypoxic stress.

To investigate the impact of tryptophan supplementation on short-chain fatty acids in the gut microbiota metabolites of hypoxia-stressed mice, we analyzed cecal contents using gas chromatography. Tryptophan supplementation significantly increased the levels of acetic acids, propionic acids, butyric acids, and total short-chain fatty acids in the gut microbiota of hypoxia-stressed mice (*p* < 0.05) (Fig. 1N–i, ii). Short-chain fatty acids, produced by the gut microbiota through metabolism, support intestinal epithelial cells and promote the repair and regeneration of the intestinal barrier.^5^ This suggests that tryptophan's enhancement of short-chain fatty acid production may be a key mechanism by which it alleviates hypoxia-induced gut microbiota dysbiosis and supports the recovery of intestinal injury.

Acute hypoxic stress induces intestinal injury, and the present study demonstrated that tryptophan could enhance intestinal function and ameliorate intestinal stress injury. Tryptophan improves the intestinal barrier and intestinal permeability by reducing acute hypoxic stress-induced damage in duodenal and colonic tissues and apoptosis of intestinal epithelial cells. Tryptophan also regulates the gut microbiota structure, increases species diversity, and promotes the growth of beneficial bacteria, thereby increasing the production of short-chain fatty acids, which support the repair and regeneration of the intestinal barrier (Fig. 1O). The above studies suggest that tryptophan attenuates hypoxic stress-induced intestinal injury by modulating the intestinal barrier and permeability and that the integrity and stability of the gut microbiota play key roles in the recovery from intestinal injury, which may be a potential mechanism for the protective effects of tryptophan against acute hypoxic stress-induced intestinal injury.

## CRediT authorship contribution statement

**Jianhua Zheng:** Conceptualization, Data curation, Formal analysis, Methodology, Visualization, Writing – original draft, Writing – review & editing. **Jingqing Chen:** Conceptualization, Formal analysis, Funding acquisition, Project administration, Supervision, Writing – review & editing. **Wensheng Zhang:** Data curation, Writing – review & editing. **Yunpeng Wu:** Data curation, Writing – review & editing. **Tongtong Qin:** Data curation, Writing – review & editing. **Yunzhi Fa:** Writing – review & editing, Data curation. **Qianyan Dong:** Data curation, Writing – review & editing. **Rui Zhang:** Data curation, Formal analysis, Supervision, Writing – review & editing. **Yefeng Qiu:** Data curation, Formal analysis, Supervision, Writing – review & editing.

## Ethics declaration

All experimental operations were in accordance with animal welfare ethics and approved by the Committee for the Management and Use of Laboratory Animals of the Military Medical Research Institute (IACUC-DWZX-2022- 049). All mice were kept in the barrier facility environment of the Experimental Animal Center of the Military Medical Research Institute (SYXK (Military) 2017-0022) at a constant temperature of 23 °C ± 2 °C, relative humidity of 40%–60%, and alternating light and dark time of 12 h/12 h, housed in plastic mouse cage (3 mice per cage; L × W × H: 403 mm × 165 mm × 175 mm), and fed and watered freely. All equipment, bedding, and feed were autoclaved before use.

## Data availability

The datasets generated during and/or analyzed during the current study are available from the corresponding authors upon reasonable request.

## Funding

This work was supported by the Specialized Scientific Research Projects for Military Laboratory Animals (China) (No. SYDW_KY[2021]06) and the Military Medical Research Institute Young Talent Fund Program (China) (No. AMMS-QNPY-2022-019).

## Conflict of interests

The authors declared no conflict of interests.

## Figures and Tables

**Figure S1:**
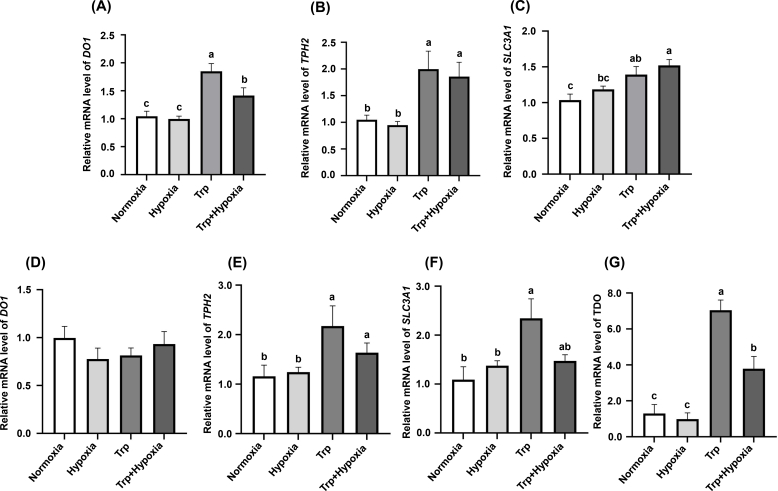
The Effect of tryptophan on kynurenine and 5-hydroxytryptamine metabolic pathways in duodenal and colonic tissues of hypoxia-stressed mice.
