# Supplementary material for: Tryptophan attenuates acute hypoxic stress-induced intestinal injury through the modulation of intestinal barrier integrity and gut microbiota homeostasis
Source: Genes Dis. 2025 Apr 4;12(6):101627. doi: 10.1016/j.gendis.2025.101627 (PMC12301910; doi:10.1016/j.gendis.2025.101627)
Supplement: Multimedia component 1 [file mmc1.docx]

**Experimental Methods**

**1 Experimental grouping and tissue sample collection**

Sixty male 7-week-old SPF-grade BALB/c experimental mice began formal experiments after 1 week of acclimatization. Using a randomized numerical table method, mice were divided into four groups: normoxia group (Normoxia), hypoxia stress group (Hypoxia), tryptophan-supplemented group (Trp), and tryptophan-supplemented and hypoxia stress group (Trp + Hypoxia), with 15 animals in each group. Mice in the normoxic and hypoxic stress groups were fed a basal diet, while mice in the tryptophan-supplemented groups were fed a tryptophan-supplemented diet. Mice in the normoxic group were kept in a central SPF barrier environment, and mice in the hypoxic group were placed in a hypoxic chamber within the barrier environment laboratory with an oxygen concentration set at 10% for 72 hours.
 At the end of the experiment, mice were fasted for 6 hours, weighed, and anesthetized with 1% pentobarbital sodium at a dose of 5 μL/g of body weight by intraperitoneal injection, and then euthanized by the neck-breaking method. After dissection, duodenum and colon tissues were collected, washed with pre-cooled PBS buffer, dried with absorbent filter paper, then flash-frozen in liquid nitrogen, and stored at -80°C for subsequent molecular index assays. Another small piece of duodenal and colon tissues was fixed in 4% paraformaldehyde for 24 hours, washed three times with saline, and preserved in 70% ethanol for subsequent production of tissue sections. To reduce individual differences, the same site was chosen for the collection of all types of tissues.

**2 Experimental feeds**

Corn-soybean meal-type basal diets were used. The tryptophan content in the basal diets produced in our laboratory usually ranged from 0.15%-0.25%. Based on pre-experimentation and literature, the optimal amount of tryptophan supplementation was determined to be 0.4%. L-tryptophan (with a purity greater than 98%) was added to the basal feed to produce supplemental tryptophan feed. The content of tryptophan in the basal feed as well as in the supplemental tryptophan feed for mice was determined, along with the feed nutrients and other amino acid contents. The tryptophan content in the basal feed for this experiment was 0.20%, and the content in the supplemental tryptophan feed was 0.40%. Details are provided in the table below:

Table 1 Amino acid content of feed

| Ingredient | Basic feeds | Supplemental tryptophan feeds |
| --- | --- | --- |
| Glycine | 0.96% | 0.90% |
| Alanine | 0.96% | 0.92% |
| Serine | 0.87% | 0.82% |
| Proline | 1.38% | 1.28% |
| Valine | 0.90% | 0.85% |
| Serine | 0.74% | 0.70% |
| Isoleucine | 0.75% | 0.70% |
| Leucine | 1.49% | 1.41% |
| Asparagine | 1.83% | 1.71 |
| Lysine | 1.06% | 0.96% |
| Glutamate | 3.70% | 3.46% |
| Methionine | 0.20% | 0.23% |
| Histidine | 0.57% | 0.51% |
| Phenylalanine | 0.93% | 0.87% |
| Arginine | 1.37% | 1.21% |
| Serine | 0.61% | 0.54% |
| Cysteine | 0.25% | 0.26% |
| Total 17 amino acids | 18.57% | 17.33% |
| Tryptophan | 0.20% | 0.40% |

**3 Blood Tests**

Mice were euthanized to collect 1 mL of blood by eyeball removal, of which 150 μL of whole blood was required. The collected blood was immediately placed into anticoagulation tubes, shaken, and mixed to prevent coagulation. The whole blood in the anticoagulation tube was then analyzed using the automatic five types of animal blood cell analyzer according to standard operating procedures to perform routine blood tests.

**4 Tissue Sections and Hematoxylin-Eosin Staining**

Fixed duodenal and colonic tissues underwent graded ethanol dehydration (75%, 85%, 90%, 95%, and 100%), followed by xylene clearing, dip waxing, and embedding. The tissues were sectioned to a thickness of 5 μm. After mounting the sections on slides, they were baked in an oven at 37°C for 48 hours. Sections were then deparaffinized to water, sequentially placed in xylene I and II for 20 minutes each, anhydrous ethanol I and II, and 75% ethanol for 5 minutes each, and washed with tap water. The sections underwent hematoxylin and eosin staining: Hematoxylin staining for 3-5 minutes, followed by eosin for 5 minutes, and finally dehydrated and sealed. The stained sections were observed under a light microscope and photographed, with the length of duodenal villi and crypt depth measured using Image J software.

**5 Scanning Electron Microscope Observation of Sample Surface Morphology**

To minimize mechanical damage and ensure freshness, tissue sampling was completed within 1-3 minutes. Duodenal samples were gently rinsed in 0.1 M PBS buffer (pH=7.4) and then quickly fixed in 2.5% glutaraldehyde for 2 hours at room temperature, followed by refrigeration at 4°C. After further PBS rinses, samples were fixed in 1% osmium tetroxide prepared in PBS buffer for 1-2 hours, avoiding light exposure. Following another set of PBS rinses, tissues underwent gradient ethanol dehydration and isoamyl acetate replacement, each for 15 minutes. The samples were then dried in a critical point dryer, mounted on double-sided adhesive with conductive carbon film, coated with gold in an ion sputtering apparatus for about 30 seconds, and observed under a scanning electron microscope.

**6 Tunel staining**

Apoptosis of intestinal epithelial cells was assessed using a TMR TUNEL Apoptosis Detection Kit. Prepared duodenal paraffin sections were deparaffinized, treated with proteinase K at 37°C for 22 minutes, and subjected to 0.1% Triton X-100 for membrane permeabilization for 20 minutes. Sections were incubated with TdT enzyme solution and a fluorescent labeling solution at 37°C for 2 hours. Nuclei were re-stained with DAPI (1:1000 dilution), and apoptosis-positive cells were observed and photographed using a Nikon imaging system DS-U3.

**7 ELISA**

Mouse serum corticosterone content was detected using competitive ELISA according to the kit instructions. First, the standard working solution, washing working solution, and HRP enzyme conjugate working solution were prepared (10 minutes before use). Then, 50 μL of the standard and serum samples that were to be tested were added to the designated wells on the enzyme plate, including blank wells. Immediately afterward, 50 μL of HRP enzyme conjugate working solution was added to each well. The plates were then incubated at 37℃ for 60 minutes, shielded from light. Following the incubation, the plate was immersed with 350 μL of washing solution and washed for 1 minute × 5 times. Next, 90 μL of substrate solution was added to each well and the plates were incubated again at 37℃ for 15 minutes, protected from light. The color reaction was terminated, and the optical density (OD) was measured at a 450 nm wavelength using an enzyme marker (detection was completed within 5 minutes).

Serum Intestinal Fatty Acid Binding Protein (IFABP) was detected according to the kit instructions. The standard working solution, washing working solution, biotinylated antibody working solution, and HRP enzyme conjugate working solution were prepared (also 10 minutes before use). Then, 100 μL of the standard and serum samples that were to be tested were added to the wells on the enzyme plate, including a blank well. The plates were incubated at 37℃, avoiding light, for 90 minutes. After this incubation, the liquid was shaken out from the wells, and 100 μL of biotinylated antibody working solution was added, followed by another incubation for 60 minutes at 37℃, shielded from light. The plates were then immersed with 350 μL of washing solution, washing them for 1 minute × 3 times, and 100 μL of HRP enzyme conjugate working solution was added to each well. Another incubation for 30 minutes at 37℃, protected from light, followed. The plates were washed for 1 minute × 5 times; 90 μL of substrate solution was added to each well, incubated at 37℃, avoiding light, for 15 minutes, then 50 μL of termination solution was added to each well to stop the color reaction. Finally, the optical density (OD) was measured with an enzyme labeling instrument at a 450 nm wavelength (detection was completed within 5 minutes).

**8 Intestinal permeability assay**

Before the experiment concluded, six mice from each group were fasted for 6 hours to ensure no excess food in the intestines would affect the absorption of large fluorescent molecules by the intestinal wall. Then, FITC-dextran (4 kD) solution, prepared in PBS and protected from light, was administered at a concentration of 40 mg/mL. Each mouse received a dose based on body weight (about 250 μL at 40 mg/100 g body weight). Three hours after gavage, the mice were anesthetized with 1% pentobarbital sodium, and eyeball extraction was performed for blood sampling. A one-time collection of 0.8 mL of blood was used to assay FITC-dextran concentration in serum. After blood collection, a skillful dislocation of the neck was performed to euthanize the animals. The serum was separated, and the FITC-dextran used for gavage was diluted in PBS starting at a concentration of 500 μg/mL, undergoing a 2-fold serial dilution across 15 gradients. Absorbance values at 525 nm with 485 nm excitation light were measured concurrently to create a standard curve, which was used to calculate the original concentration and take the average value.

**9 Fecal DNA extraction, amplicon library preparation, and sequencing**

Fecal DNA extraction: Feces from four groups of mice, Normoxia, Hypoxia, Trp, and Trp + Hypoxia, were collected with sterilized tweezers before dissection. Each mouse feces sample (30-50 mg) was transferred to a 2 mL centrifuge tube, mixed with 0.25 g of glass beads, and vortexed to thoroughly disrupt the feces. The samples were then incubated at 70°C for 10 minutes, centrifuged at 12,000 rpm for 5 minutes, and the supernatant was collected. Approximately 500 μL of the clarified middle layer liquid was aspirated into a sterile centrifuge tube, and the DNA extraction process was completed with various washing and binding steps using proprietary buffer solutions (PB31, BB31, CB31, WB31) and magnetic beads. Care was taken to avoid aspirating the beads. After drying the beads at room temperature for 5-10 minutes post-ethanol evaporation, 80 μL of Elution Buffer (EB) was added, vortexed for 30 seconds, and incubated at 70°C for 5 minutes. The solution was placed on a magnetic rack until clear, and the supernatant was transferred to a new sterile centrifuge tube. The extracted fecal DNA was stored at -80°C.

Amplicon library preparation and sequencing: Quality control checks were performed according to the sample and product requirements. PCR amplification was conducted using a prepared fusion primer reaction solution, utilizing the previously prepared DNA. The appropriate PCR conditions were set for DNA amplification. Library testing was conducted to ensure quality and qualified libraries were sequenced using the designated Illumina sequencing platform.

**10 16S sequencing analysis**

For data filtering, reads matching the primers were first processed using the software Cutadapt v2.6. Primer and splice contaminations were removed using a window length of 30 bp; if the average quality value within this window was lower than 20, the end sequence of the reads was removed. Reads retaining more than 75% of their original length were kept. Reads containing Ns and low-complexity reads (i.e., reads with 10 consecutive ATCG occurrences) were also removed to finally obtain clean data.

For tags concatenation and OTU clustering, the Usearch method was used to cluster OTUs based on 97% sequence similarity. The reads (double ends) obtained from sequencing were assembled into one sequence by FLASH (sequence splicing software) to obtain the highly variable region of tags (minimum match length: 15 bp and mismatch rate of overlapping regions: <10%). Subsequently, OTU clustering (at 97% similarity) was performed using USEARCH. UCHIME was then used to remove chimeras generated by PCR amplification from the representative sequences of OTUs. Finally, USEARCH_GLOBAL compared tags with OTUs, and the abundance statistics table of OTUs was analyzed.

Species annotation was performed using RDP (classifier software) against a database with a confidence threshold of 0.6. Filtering of the annotation results involved the removal of unannotated OTU sequences and the exclusion of sequences that do not belong to the species under study.

**11 Determination of short-chain fatty acids in the contents of the cecum**

The content of short-chain fatty acids (SCFA) in cecum contents was determined using gas chromatography. Approximately 50 mg of cecum contents was weighed and homogenized in 1.0 mL of water containing 0.5% phosphoric acid and 50 μg/mL 2-ethylbutyric acid for 30 minutes in an ice-water bath, then transferred to 4 ℃ for 30 minutes. The mixture was then centrifuged for 15 minutes at 13,000 rpm at 4 ℃ to obtain the supernatant. Free SCFA was extracted with ethyl acetate in an ice-water bath for 10 minutes. The sample was centrifuged again (13,000 rpm for 10 minutes at 4 °C). Determination was performed using an Agilent 8890 B gas chromatography-mass spectrometer (GC-MS) system equipped with an HP-FFAP column. High-purity helium was used as the carrier gas (flow rate of 1.0 mL/min, split ratio of 10:1). The heating program started with an initial column incubator temperature of 80 ℃, increased from 120 ℃ to 200 ℃, and held at 230 ℃ for 3 minutes. The content of SCFAs in the cecum contents was detected by the GC-MS, and concentrations of various SCFAs were calculated according to the standard curves, and the concentration of various SCFAs per gram of the samples was calculated based on the mass and total volume of the samples.

**12 Tissue RNA extraction, real-time fluorescence quantitative PCR analysis**

Remove 30-50 mg of tissue from liquid nitrogen cryopreservation. Add 1 mL of TRIGent and homogenize and lyse the sample at low temperature, following the specific steps outlined in the TRIGent reagent kit for RNA extraction. Once the RNA precipitate is obtained, centrifuge it at 12,000 r/min for 15 min at 4 ℃, then discard the supernatant. Wash the RNA precipitate with 1 mL of 75% ethanol containing 0.1% DEPC, and repeat this washing step once more to enhance the RNA quality. Afterward, centrifuge again at 12,000 r/min for 5 min at 4 ℃, discard the supernatant, and dry the RNA precipitate for 1-2 min at room temperature. Subsequently, solubilize the RNA in DEPC-treated water. Determine and record the concentration and quality of the RNA using a spectrophotometer. According to the instructions of the reverse transcription kit, take 2 μg of the total RNA, remove genomic DNA, and perform a reverse transcription reaction to obtain cDNA

The real-time quantitative PCR reaction was performed using the SYBR Green dye method with a reaction system of 12.5 μL. The cycling parameters included denaturation at 95 ℃ for 30 s and 1 cycle; denaturation at 95 ℃ for 5 s, annealing at 60 ℃ for 30 s, and amplification for 40 cycles, and continuous fluorescence measurements were applied with the melting curve program. The relative expression level of the target gene was calculated using 2^-ΔΔCt^ with *β-actin* as the internal reference gene, and the primer sequences are shown in Table 2.

Table 2 Sequence list of qRT-PCR primers

| Gene | Forward primer（5ʹ→3ʹ） | Reverse primer（5ʹ→3ʹ） |
| --- | --- | --- |
| *β-Actin* | GGCTGTATTCCCCTCCATCG | CCAGTTGGTAACAATGCCATGT |
| *SLC3A1* | ATGAAGGGATGCCGAACCAAT | CAGGGATACTCACGGCGTTG |
| *IDO1* | GCCTCCTATTCTGTCTTATGCAG | ATACAGTGGGGATTGCTTTGATT |
| *TDO* | ATGAGTGGGTGCCCGTTTG | GGCTCTGTTTACACCAGTTTGAG |
| *TPH2* | GGTTGTCCTTGGATTCTGCTG | GCCTGGATTCGATATGAAGCAT |

**13 Measurement of serum free amino acid concentration**

The determination of free amino acid content in serum was carried out according to the method recorded in the national standard (GB/T 30987-2020). Firstly, 0.1 mol/L hydrochloric acid solution, 50% acetonitrile solution, mixed amino acid standard reserve solution, mixed amino acid standard working solution and mixed amino acid standard intermediate solution were prepared. Then the mobile phases were prepared, 200 mmol/L ammonium formate solution, 20 mmol/L ammonium formate-water solution (mobile phase A), 20 mmol/L ammonium formate-acetonitrile solution (mobile phase B) were prepared. The extract was transferred to a 10 mL volumetric flask, and then diluted by adding 50% acetonitrile solution and was fixed to the scale, gently mixed, 2 mL of the diluted solution was taken and centrifuged at 4 ℃ for 10 min at 14 000 r/min, and finally the supernatant was taken and determined by HPLC.

**14 Statistical analyses**

The values of each experimental result were expressed as mean ± standard error ($\overline{x}$ ± *s*$\overline{x}$). Statistical analysis was performed using SPSS 26.0 software, and comparative data analysis between groups was conducted using the one-way ANOVA method, with *P*<0.05 considered significant. Graphs were generated using GraphPad Prism 9.0 software.
