# Supplementary material for: Tryptophan attenuates acute hypoxic stress-induced intestinal injury through the modulation of intestinal barrier integrity and gut microbiota homeostasis
Source: Genes Dis. 2025 Apr 4;12(6):101627. doi: 10.1016/j.gendis.2025.101627 (PMC12301910; doi:10.1016/j.gendis.2025.101627)
Supplement: Multimedia component 2 [file mmc2.docx]

Supplementary Table 1: Effect of tryptophan on serum free amino acid concentration in mice subjected to acute hypoxic stress.

| Amino acid  μg/ml | Groups | | | | *P*-Value |
| --- | --- | --- | --- | --- | --- |
|  | Normoxia | Hypoxia | Trp | Trp+Hypoxia |  |
| Glycine | 9.53±0.49^a^ | 8.50±0.37^b^ | 10.51±0.66^a^ | 9.95±0.39^a^ | <0.05 |
| Alanine | 17.06±1.18^a^ | 11.04±0.98^b^ | 20.79±1.63^a^ | 22.06±1.29^a^ | <0.01 |
| Serine | 5.09±0.23^b^ | 4.26±0.15^bc^ | 6.02±0.36^ab^ | 6.64±0.20^a^ | <0.0001 |
| Proline | 4.21±0.25^b^ | 3.28±0.19^b^ | 5.48±0.42^a^ | 5.11±0.24^a^ | <0.01 |
| Valine | 8.86±0.34 | 10.69±0.79 | 11.91±0.99 | 8.75±0.73 | 0.106 |
| Serine | 6.29±0.32^b^ | 5.16±0.34^b^ | 8.31±0.48^a^ | 6.10±0.32^b^ | <0.01 |
| Isoleucine | 4.77±0.25^b^ | 7.38±0.69^a^ | 8.01±0.85^a^ | 5.72±0.54^a^ | <0.05 |
| Leucine | 8.09±0.36^b^ | 11.96±0.96^a^ | 13.51±1.38^a^ | 10.51±0.80^a^ | <0.05 |
| Asparagine | 1.12±0.07^a^ | 0.55±0.07^b^ | 1.58±0.23^a^ | 1.58±0.11^a^ | <0.01 |
| Lysine | 8.53±0.47^a^ | 6.38±0.2^b^ | 11.07±0.96^a^ | 10.52±0.51a | <0.01 |
| Glutamate | 4.87±0.35^b^ | 4.69±0.27^b^ | 8.31±0.6^a^ | 9.37±0.55^a^ | <0.01 |
| Methionine | 21.21±0.55^a^ | 14.22±0.47^c^ | 26.74±1.88^a^ | 19.95±0.69^b^ | <0.01 |
| Histidine | 3.56±0.35 | 3.83±0.09 | 3.90±0.33 | 4.08±0.33 | 0.715 |
| Phenylalanine | 6.38±0.21^b^ | 8.70±0.35^a^ | 8.81±0.59^a^ | 7.08±0.41^a^ | <0.01 |
| Arginine | 3.37±0.37^a^ | 2.86±0.12^b^ | 4.83±0.47^a^ | 3.67±0.66^a^ | <0.01 |
| Serine | 5.85±0.24^a^ | 3.60±0.24^b^ | 5.49±0.38^a^ | 4.93±0.14^a^ | <0.01 |
| Cysteine | 0.15±0.02^ab^ | 0.20±0.02^a^ | 0.14±0.01^b^ | 0.19±0.01^a^ | <0.05 |
| Tryptophan | 9.44±0.31^b^ | 11.61±0.54^a^ | 12.39±0.62^a^ | 10.85±0.49^ab^ | <0.05 |

**Table Legends:**

Supplementary Table 1: Data are expressed as mean ± standard error of the mean, different letters in the table indicate significant differences (*P*<0.05).
